# Are Drinking Motives Universal? Characteristics of Motive Types in Alcohol-Dependent Men from Two Diverse Populations

**DOI:** 10.3389/fpsyt.2018.00038

**Published:** 2018-02-13

**Authors:** Verena Ertl, Melissa Preuße, Frank Neuner

**Affiliations:** ^1^Clinical Psychology and Psychotherapy, Department of Psychology, Bielefeld University, Bielefeld, Germany; ^2^vivo international, Konstanz, Germany

**Keywords:** addiction, alcohol use disorder, drinking motives, reward- and relief-drinking, self-medication, trauma, childhood maltreatment, mental health

## Abstract

**Background and Aims:**

Since alcohol use disorders are among the most prevalent and destructive mental disorders, it is critical to address factors contributing to their development and maintenance. Drinking motives are relevant driving factors for consumption. Identifying groups of drinkers with similar motivations may help to specialize intervention components and make treatment more effective and efficient. We aimed to identify and describe distinct motive types of drinkers in dependent males from two diverse cultures (Uganda and Germany) and to explore potential differences and similarities in addiction-related measures. Moreover, we investigated specific links between motive types and childhood maltreatment, traumatic experiences, and symptoms of comorbid psychopathologies.

**Methods:**

To determine distinct drinking motive types, we conducted latent class analyses concerning drinking motives (Drinking Motive Scale) in samples of treatment-seeking alcohol-dependent men (*N* = 75). Subsequently we compared the identified motive types concerning their alcohol consumption and alcohol-related symptoms (Alcohol Use Disorders Identification Test), history of childhood maltreatment (Childhood Trauma Questionnaire), trauma exposure (Violence, War and Abduction Exposure Scale), psychopathology (Posttraumatic Stress Diagnostic Scale, Depression-section of the Hopkins Symptom Checklist, and Brief Symptom Inventory) and deficits in emotion regulation (Difficulties in Emotion Regulation Scale).

**Results:**

We found two congruent drinking motive types in both contexts. Reward-oriented drinking motives like the generation of positive feelings and enhancing performance were endorsed almost equally by both motive types, whereas high relief motive endorsement characterized one group, but not the other. The relief motive type drank to overcome aversive feelings, withdrawal, and daily hassles and was characterized by higher adversity in general. Emotional maltreatment in childhood and psychopathological symptoms were reported to a significantly greater extent by relief drinkers (effect sizes of comparisons ranging from *r* = 0.25 to *r* = 0.48). However, the motive types did not differ significantly on alcohol consumption or alcohol-related symptoms and traumatic experiences apart from childhood maltreatment.

**Conclusion:**

The chronology of addiction development and patterns of drinking motivation seem to be similar across cultures, i.e., that motive targeting interventions might be applicable cross-culturally. Addressing comorbid symptomatology should be a key treatment component for relief drinkers, whereas finding alternatives for the creation of positive feelings and ways to counteract boredom and inactivity should be a general treatment element.

## Introduction

Globally, alcohol abuse and dependency is one of the key causes of premature illness and death, loss of functionality and productivity, social decline, delinquency, and domestic and community violence (1, 2). Alcohol consumption is a prevalent worldwide phenomenon, and alcohol-related deterioration of physical and mental health is a highly relevant individual and societal threat. It is not surprising, then, that researchers have been interested in studying factors causing or contributing to the initiation and maintenance of drinking, both in general and in specific situations. Several conceptually related constructs and terminologies have been coined during the search to answer these questions.

One such construct, the self-medication hypothesis, describes the use of drugs to alleviate or suppress suffering as one attempt to cope with stressors as well as psychopathological symptoms and the related negative affective and physiological states (3). When self-medication is successful, the consumption of alcohol is negatively reinforced. Therefore, the hypothesis provides an explanation for both the onset and maintenance of drinking. Similar predictions can be made following most of the past decades’ literature. This includes, for instance, early models of drug motivation by Wikler (4) or the tension-reduction theory (5)—a combination of Hull’s (6) drive-reduction theory of reinforcement with the tension-relieving effect of alcohol—or Solomon’s opponent-process model (7) that focuses primarily on withdrawal relief.

A more recent model, the affective processing model (8), borrows features of these earlier models, but focuses on the role of negative affect as the motivationally predominant element of consciously and unconsciously triggered consumption. It states that, at low levels of negative affect, consumption tends to be automatic or proceduralized. At moderate levels, controlled processing is possible, i.e., expectancies play a role in influencing consumption, whereas hot information processing (9) precludes cognitive control at high levels of negative affect. Moreover, consumption may not be prompted solely by negative affect or produced by withdrawal but also by stressful events or internal states associated with negative affect (8).

Models like these seem to be supported by high comorbidity rates between substance-related disorders and depressive, anxiety, and trauma and stressor-related disorders [e.g., Ref. (10–18)]. Furthermore, cross-sectional and longitudinal studies (including experience sampling studies) report a link between the onset of negative affect or symptoms of mental health disorders and alcohol consumption or alcohol-related symptoms [e.g., Ref. (19–29)]. However, these models have neglected the potential presence of other motivators to drink apart from coping with burdens and negative affect. Cox and Klinger’s (30) motivational model of alcohol use additionally suggests consumers drink for exhilarative and social reasons. This means that consumption may not only be driven by negative reinforcement but also by positive reinforcement. Drinking to enhance is an appetitive process, which, when successful, reinforces the consumption of alcohol and may foster the development of dependence, similar to what occurs during negative reinforcement.

According to Cox and Klinger (30), positive and negative reinforcement can be further conceptualized as being internally versus externally generated. For instance, a drinker can pursue the internally generated reward of augmenting his or her performance or the externally generated reward of positive social interactions. Likewise consuming alcohol may serve the goal of avoiding social exclusion (external) or attenuating negative emotions (internal) (30). Cooper and colleagues (31–33), who followed Cox and Klinger’s (30) model, consequently described the motivations along four dimensions: enhancement, social, coping, and conformity motives. Other researchers follow a similar logic but differentiate among positive reinforcement, negative reinforcement, and obsessive craving (34–36). Similar to Wills and Shiffman (37), they describe in their psychobiological model of craving for alcohol that reward drinkers aim at the stimulating and relief drinkers at the anxiety- or stress-attenuating properties of alcohol. That is, relief drinkers strive to attenuate overarousal and reward drinkers strive to elevate their state of underarousal. This is partly in contrast to other researchers, who claim that positive affect precedes enhancement drinking (33). Cloninger et al. (38) share the same idea that one type of dependent drinkers use alcohol mainly to relieve anxiety and another type to induce euphoria. However, he describes further features to characterize his prototypes of type I and type II alcoholism. The former is reported to be additionally linked to high harm avoidance, low novelty seeking, onset after 25 years of age, and genetic and environmental contributing factors. The latter is described to affect men more often than women, to be associated with high novelty seeking, onset before 25 years of age, and to depend primarily on genetic predisposition.

Another concept closely related to drinking motives is drinking expectancy (39). Expectancies are defined as beliefs concerning the probability that drinking will produce certain effects. This is in contrast to motives, which are defined as desired outcomes that an individual hopes to achieve by drinking. Leigh (40) found that an alcohol user must endorse a specific expectancy to achieve a certain effect before consumption, but that he or she will not necessarily drink to achieve this specific effect simply because the corresponding expectancy is endorsed. In other words, it is possible to hold expectations about the effects drinking will have, but that does not necessarily mean that an individual will then proceed to drink to fulfill those expectancies. According to the cognitive model of alcohol use (41), the activation of expectancies precedes the actualization of drinking motives. Consequently, drinking motives are the more proximal and diagnostic factors of actual alcohol use: they are the gateway through which more distal influences (like alcohol expectancies) are mediated and thus should be the target of interventions (32, 33, 42–44). Although slightly different in conceptualization and focus, each of the models and theories discussed ultimately strive for the same goal, as far as treating addiction is concerned: to understand the driving factors of consumption to open doors for controlling consumption and delivering valuable information for the fine tuning of intervention programs.

Since drinking typically starts in adolescence, an abundance of research has focused on drinking motivations in young samples of high-income countries, with the aim being to understand what makes them start and continue drinking [e.g., Ref. (25, 31, 43, 45–53)]. Comparatively little research has been conducted around drinking motives in adults (33, 54–57) and more specifically in dependent drinkers, with the exception of studies researching self-medication in addicts with a PTSD, depression, or anxiety comorbidity (58–61). To the best of our knowledge, no study on drinking motivation thus far has included dependent drinkers from low-income or postconflict settings.

According to the global status report on alcohol and health (62), Uganda is among the top six countries in Africa concerning male per capita intake of alcohol (14.4 l of pure alcohol in males older than 14 years). Considering the per capita intake of drinkers, Uganda ranks 24th worldwide, indicating that there is a considerable percentage of abstainers, but that those who do drink consume extremely high amounts (23.7 l of pure alcohol). Germany ranks 19th in Europe in male per capita intake of alcohol (16.8 l of pure alcohol in males older than 14 years) and 83rd worldwide concerning per capita intake of drinkers only (14.7 l of pure alcohol). Consequently, it is not surprising that Uganda has been reported as having the highest prevalence of negative consequences of alcohol consumption on a personal and social level (physical, psychological and financial, work related, and social functioning) in a 26-country comparison study including countries with varying income levels from all continents (63, 64).

The current literature on drinking motivation implies that relief and reward drinking or enhancement, coping, social, and conformity drinking are correlated, suggesting that drinking motives are dynamic and can change intraindividually. In addition, individuals can follow several drinking motives concurrently even within specific situations (27, 32, 33, 43, 45, 49, 58, 59, 65–68). Therefore, we assumed that a person-centered analytic approach like latent class analysis (LCA) would suit the current knowledge on drinking motivation better than factor analytic approaches. We hypothesized that the LCA would not reveal pure enhancement versus pure coping motive types. Further, in contrast to studies conducted in non-dependent samples (69), we assumed that all resulting motive types, no matter how characterized, would show similar alcohol use, but not equally severe alcohol-related psychopathology. Symptoms of alcohol-related and addictive disorders should be more pronounced the more coping motivation is present.

We additionally expected that the individuals especially characterized by coping motives would start drinking earlier, consequently report higher chronicity of alcohol dependency, and show more symptoms of other mental health disorders. Accordingly, we assumed that early aversive experiences would predict class membership, i.e., relief drinkers should report more experiences of childhood maltreatment in their families of origin. From a clinical point of view, these aspects are of particular interest since they clarify whether patients with alcohol use disorders reporting specific motives or motive constellations differ on therapeutically relevant measures.

## Materials and Methods

### Participants and Procedures

The German sample consisted of alcohol-dependent men (*n* = 49) recruited from wards specialized in the treatment of substance-related and addictive disorders at two psychiatric hospitals. The Ugandan participants were alcohol-dependent men (*n* = 26) living in Gulu, who were interested in taking part in an inpatient treatment program that was announced *via* the radio and a community-based organization called Program for Prevention, Awareness, Counseling and Treatment of Alcoholism (PACTA). Alcohol-related diagnostic status was recorded according to the ICD-10 by the practitioners routinely charged with the task of diagnosing and treating dependent patients in the respective wards at treatment entry (Clinic of Psychiatry and Psychotherapy, Ev. Hospital Bielefeld; Lippische Nervenklinik, Bad Salzuflen and Gulu Regional Referral Hospital, Gulu).

We chose to exclude female participants, since there were only two persons who expressed an interest in participation in the Ugandan sample. Characteristics of the two samples are summarized in Table 1.

**Table 1:** Level of trauma exposure, indices of alcohol consumption, and psychopathological symptoms in the German and Ugandan samples (means and SDs are reported).

	German sample (*n* = 49)	Ugandan sample (*n* = 26)	Effect size (*r*)^a^	*p* value
Age	42.9 (11.8)	39.1 (9.6)	0.15	0.19
**Trauma exposure**				
*Childhood maltreatment*				
Physical maltreatment^b^	2.0 (2.3)	3.3 (1.5)	0.39	< 0.001
Emotional maltreatment^b^	3.3 (3.3)	3.6 (2.3)	0.13	0.27
*Traumatic events (VWAES)*				
Traumatic event types, experienced^c^	–	4.7 (2.1)		
Traumatic event types, witnessed^c^	–	6.3 (2.7)		
Traumatic event types, perpetrated^d^	–	0.4 (1.0)		
Age at worst traumatic event	–	28.2 (11.3)		
**Alcohol-related indices**				
First consumption (age)	14.2 (3.8)	14.4 (5.9)	0.09	0.44
Start of dependency (age)	29.9 (10.3)	28.1 (9.0)	0.07	0.58
Chronicity of dependency (years)	14.6 (10.7)	10.9 (7.7)	0.13	0.30
Standard-consumption on a typical drinking day^e^	17.9 (13.0)	22.3 (13.0)	0.17	0.14
**Psychopathology**				
Alcohol-related symptoms (AUDIT)^f^^,^^g^	22.4 (8.2)	27.7 (6.9)	0.31	0.005
PTBS symptoms (PDS)^h^	–	2.0 (4.6)		
Depression symptoms (DHSCL)^i^	–	2.1 (0.7)		
General psychopathology (BSI), GSI score^j^	1.1 (0.7)	–		
Emotion dysregulation (DERS)^k^	89.6 (25.9)	–		

*If not otherwise indicated, results are based on nonparametric Mann–Whitney tests*.

*^a^Following Cohen’s suggestion, effect sizes of r = 0.10 can be interpreted as small effect, r = 0.30 as medium effect, and r = 0.50 as large effect*.

*^b^Scale range: 0–10*.

*^c^Scale range: 0–11*.

*^d^Scale range: 0–6*.

*^e^One standard drink contains 13 g of pure ethanol*.

*^f^Scale range: 0–40*.

*^g^Results are based on a t-test*.

*^h^Scale range: 0–51*.

*^i^Scale range: 1–4*.

*^j^Scale range: 0–4*.

*^k^Scale range: 36–180*.

*AUDIT, Alcohol Use Disorders Identification Test; BSI, Brief Symptom Inventory; DERS, Difficulties in Emotion Regulation Scale; DHSCL, Depression-Section of the Hopkins Symptom Checklist; GSI, Global Severity Index; PDS, Posttraumatic Stress Diagnostic Scale; VWAES, Violence, War and Abduction Exposure Scale; –, not assessed*.

German participants were recruited through informational flyers that were distributed in the wards. In the event an individual was interested in participating, he received more detailed information in written form about the content of the study and its aims, about its voluntary and anonymous nature, and about data handling. Subsequently, participants gave written consent before filling out the self-rating instruments in paper and pencil versions. Study completion required 30 min on average. Assessments were conducted in a private room that was provided by the respective wards and took place as early as possible in the patients’ treatment periods. A study assistant was always present in the event that any participant had questions regarding the study.

Due to high rates of illiteracy in the Ugandan sample, seven local counselors affiliated with the NGO vivo international and proficient in using all screening instruments utilized in this study carried out the data collection and judged the validity of provided information. Clinical psychologists experienced in cross-cultural research and familiar with the northern Ugandan context were present at all interview times and provided supervision and training. Interviews took place on the premises of the outpatient clinic for survivors of violence and trauma in Gulu town mostly 1 week before treatment start. Questionnaires were routinely checked for missing items and inconsistencies on site. Before starting the interview, the project and procedures were explained in detail, and participants were encouraged to raise questions. Written informed consent was obtained (signature or fingerprints) from all subjects in accordance with the Declaration of Helsinki (70). Neither German nor Ugandan participants received any financial or material benefit for participating in the study. However, Ugandan participants were given a compensation of 5,000 UGX (approximately US$1.80) for their transportation costs.

Ethical approval for the studies was provided by the Ethical Committee of Bielefeld University following the guidelines of the German Psychological Society. These guidelines are in agreement with the American Psychological Association’s code of ethics. In addition, all materials and procedures have been reviewed by the Lacor Hospital Institutional Research and Ethics Committee, Gulu, and approved by the Ugandan National Council for Science and Technology.

### Measures

Versions of the screening instruments in Luo, which is the local language in northern Uganda, have been previously created and utilized. The procedure of translation and adaptation is described elsewhere (71, 72).

#### History of Childhood Maltreatment

Aversive events experienced in the respondents’ families of origin were assessed with the Childhood Trauma Questionnaire (CTQ) in the German sample (73–75). The CTQ is a 28-item event checklist with a five-point Likert-type answering format ranging from 1 (“never true”) to 5 (“very often true”). The men were answering the questions retrospectively from the time when they were growing up in their respective family of origin. Although the dimensional format was used in the German sample, we used a dichotomous format (“experienced” or “not experienced”) in the Ugandan sample for reasons of practicality. Subsequently, events answered positively were summed up per subcategory (physical abuse, emotional abuse, physical neglect, emotional neglect, and sexual abuse). In addition, a composite score for emotional maltreatment (sum of emotional abuse and emotional neglect) and physical maltreatment (sum of physical abuse and physical neglect) was created. The dimensional scaling in the German sample was dichotomized before creating sums. Ratings of 3 (“sometimes true”) to 5 (“very often true”) were re-coded as “experienced”. The German version of the CTQ is reported to be reliable and valid (75–77), and similar checklists as the one used in this study have been applied successfully in the Ugandan context (78, 79).

#### Trauma Exposure

Trauma exposure beyond childhood maltreatment was only assessed in the Ugandan sample. The Violence, War and Abduction Exposure Scale is a 34-item checklist of potentially traumatic events that was developed specifically for use in the northern Ugandan context (80, 81). It consists of 18 general event types, adapted from the Clinician-Administered PTSD Scale (82), 6 LRA-specific event types that capture events related to the rebel army (e.g., “Have you ever been forced to eat human flesh by the LRA?”), and 6 forced perpetration event types (e.g., “Have you ever been forced to kill someone by the LRA?”). The original checklist was shortened by four items related to family violence, since the previously described specific checklist was used to cover violence in the family of origin. Two additional items applied to women only (e.g., “Have you given birth to a child during captivity?”) and were also not included. Participants were asked whether a certain traumatic event had happened to them in the past, and answers were coded in a simple yes/no format. Scores of event types experienced, event types witnessed, and event types perpetrated were created by summing positive answers.

#### Alcohol Consumption and Symptoms of Alcohol Use Disorders

Alcohol consumption and alcohol-related symptoms were measured using the 10-item interview version of the Alcohol Use Disorders Identification Test [AUDIT (83)]. Items one to three assess frequency and typical quantity of alcohol consumption and frequency of heavy drinking. Items four to six determine symptoms of dependence, and items seven to ten establish harmful alcohol use. Items one to eight are coded on five-point Likert-type scales ranging from 0 to 4 with varying anchor descriptions fitting the content of the respective question. Items nine and ten offer only three anchors with scoring options 0, 2, and 4. The sum of items 1 through 10 is commonly used for score interpretation. The AUDIT identifies hazardous and harmful alcohol use and possible dependence being consistent with ICD-10 definitions. A score of 8–14 has been established as an indicator for hazardous use, a score between 15 and 19 as indicator for harmful drinking, and a score of 20 and above as indicator for dependent drinking. The AUDIT has been reported to accurately measure risk across gender, age, and cultures (84, 85). The AUDIT had already been successfully employed in northern Uganda in previous research (72, 86). Apart from the AUDIT, other alcohol-related indices were assessed in both samples. We asked for age at the time of initial consumption, as well as age at the beginning of dependency. Typical consumption on an ordinary drinking day was converted to standard drinks. One standard drink was defined as containing 13 g of pure ethanol. In Uganda, interviewers were trained to use a conversion table to be able to translate typical types and serving sizes of local alcoholic beverages into standard drinks.

#### Drinking Motives

We created a Drinking Motive Scale on the conceptual basis of Cox and Klinger’s (30) motivation model of alcohol use, assuming that drinkers follow the aim of positive or negative reinforcement with their consumption. Items were partly based on a known questionnaire, the Trierer Alkoholismusinventar (TAI; 20 items) (87). Ten further items were added to assess drinking to counteract the effects of alcohol withdrawal (four items); to complement the motive of coping with aversive feelings, images, and memories (four items); and to assess the motive to avoid current problems in life (two items). On conceptual grounds, the motive items could be grouped according to six broader concepts: (a) enhancing performance; (b) generating positive emotions; (c) enhancing social functionality; (d) counteracting aversive feelings, images, and memories; (e) counteracting withdrawal symptoms; and (f) avoiding current problems in life. The first two aim to measure positive reinforcement, and the latter three aim to measure negative reinforcement. The social motive items are mixed, with the latter three targeting negative reinforcement. The conceptual subscales are in accordance with the factors repeatedly reported for another frequently used instrument, the drinking motives questionnaire [DMQ (32)]: enhancement, coping, and social. The DMQ has been developed for and mainly used in adolescent and young adult samples [e.g., Ref. (31, 47–51, 53, 67, 68, 88–90)] and has rarely been used in adult or dependent samples [e.g., Ref. (53, 57, 58)]. We deliberately did not ask for items that address the fourth factor of the DMQ, conformity (the urge to be part of a peer group), since this factor was assumed to primarily play a role in adolescent drinking behavior and drinking onset and has been shown to be more relevant in younger adolescents (32). Since individuals in our samples presented with a history of dependence lasting for more than a decade, we did not consider drinking to get into or feel as a part of a desired group as a relevant motive in current drinking. Instead we added a subscale for drinking against withdrawal symptoms and more items targeting relief/coping motives. Our questionnaire used a dichotomous format, and participants were asked to answer the respective items according to whether the motives were met when they were consuming in the past month prior to the interview.

#### Psychopathology

PTSD symptoms were assessed in the Ugandan sample only. The Posttraumatic Stress Diagnostic Scale (91) provides measures of overall and subscale symptom severity. Its 17 items reflect the core PTSD criteria of re-experiencing, avoidance, and hyperarousal according to the DSM IV (92). Each item can be scored on a four-point Likert-type scale ranging from 0 (“not at all or only one time in the past month”) to 3 (“five or more times a week or almost always”). A validation study in northern Uganda confirmed applicability, very good internal consistency, and good correspondence with expert diagnoses of PTSD (80). When the A-criterion for PTSD was met, we calculated the sum of the 17 symptom items to obtain a measure of symptom severity.

The 15-item Depression-Section of the Hopkins Symptom Checklist [DHSCL (93)] was used in the Ugandan sample to assess the perceived intensity of symptoms of depression in the week prior to the interview. Answers are coded on a four-point Likert-type scale ranging from 1 (“the symptom bothered or distressed me not at all”) to 4 (“the symptom bothered or distressed me extremely”). The DHSCL was chosen because it had been extensively used for the assessment of symptoms of Depression across a wide variety of cultures including several East African populations [e.g., Ref. (78, 81, 94–96)]. We applied the commonly used procedure of summing up the item scores and dividing them by the number of items.

To measure the presence of psychopathological symptoms in the German sample, we used the Brief Symptom Inventory [BSI (97)]. The BSI is a 53-item screening instrument providing information on nine dimensions: somatization, obsessive-compulsity, interpersonal sensitivity, depression, anxiety, hostility, phobic anxiety, paranoid ideation, and psychoticism. Each item is rated on a five-point Likert-type scale ranging from 0 (“not at all”) to 4 (“extremely”), indicating the severity of impairment by the respective symptom in the last 7 days. We calculated the Global Severity Index (GSI) for this study, i.e., summing up the item scores and dividing them by the number of items. The psychometric quality of the German BSI version is reported to be excellent (97, 98).

#### Emotion Regulation

The Difficulties in Emotion Regulation Scale [DERS (99)] was applied in the German sample only. It consists of 36 items assessing multiple aspects of emotional dysregulation. Respondents are asked to judge how often each statement generally applies to them. Scores can be interpreted according to six subscales: non-acceptance of emotional responses, difficulties engaging in goal-directed behavior, impulse control difficulties, lack of emotional awareness, limited access to emotion regulation strategies, and lack of emotional clarity. Items are rated on five-point Likert-type scales ranging from 1 (“almost never”) to 5 (“almost always”). To reach at a score of overall emotional dysregulation, the item scores are summed up. The psychometric properties of the German version of the DERS have been described as being satisfactory (100).

### Data Analyses

Since our main interest was not grouping drinking motive items, as in variable-centered approaches like factor analysis, but grouping respondents based on the patterns of their answers concerning their drinking motives, we chose the person-centered analytic approach of LCA. Finite mixture models, like LCA, provide a framework for identifying subgroups of individuals that are not directly observable. To be able to compare response patterns of Ugandan and German alcohol dependents, we calculated two separate LCAs. The LCAs provided us with information on the probability of an individual in a certain motive class to endorse a certain drinking motive (conditional item probability, given a certain class membership) and on the relative prevalence of a certain motive class (class probability). Sample sizes for LCAs were reduced by 5 individuals in the German and by 1 individual in the Ugandan sample, due to missing information of at least 1 of the 30 motive items.

We compared models with differing numbers of classes on the values of common parsimony indices, the Akaike Information Criterion (AIC), and the Schwarz Bayesian Criterion (SBC). The AIC hinted at different solutions in the two samples, and the solution in the German sample was not parsimonious (suggesting five classes). In both samples, the SBC also referred to as Bayesian Information Criterion (BIC) produced lowest values for a two class solution. The model that produces the lowest values on parsimony indices can be judged the best model. In addition, simulation studies have indicated that commonly the BIC was the best information criterion for identifying the correct number of classes (101). Given that the BIC indicators favored a two class solution in both samples and its usefulness taking conceptual considerations into account led to our decision for the two class solution.

Subsequent comparisons of the resulting two motive types across the two diverse samples were possible for most variables, since they were assessed equally in both samples. However, psychopathology was assessed with different screening instruments; therefore, we created a composite measure of psychopathology consisting of the mean of z-transformed PTSD and depression scores in the Ugandan sample and the z-transformed global severity score of the BSI in the German sample. To test the robustness of the findings on psychopathology, we additionally compared the motive types on depression symptoms only. Since the BSI and the DHSCL have a shared history of instrument development the 6 items of the depression subscale of the BSI exactly match 6 of the 15 DHSCL items. The depression symptom score consequently combined the z-transformed scores on these six items in both samples.

Data analyses were carried out with JMP version 13.1.0 (102).

## Results

### Descriptive Statistics

The samples of treatment-seeking, alcohol-addicted men from two diverse cultures and vastly divergent living contexts did not differ in current age, age at first consumption, or age at the beginning of dependency. Consequently, they did not differ concerning the chronicity of addiction. While the typical amount of consumption in standard drinks (containing 13 g of pure ethanol) also did not differ, the Ugandan sample was significantly more affected by alcohol-related symptoms (as indicated by the AUDIT score). Both samples were equally affected by emotional maltreatment in their families of origin. However, they differed in their histories of physical maltreatment, with the Ugandan sample being affected more heavily. Further characteristics, such as traumatic events beyond childhood maltreatment and different measures of psychopathological symptoms that were only assessed in one of the two samples, are displayed in Table 1.

### Motive Types

**Figure 1:**
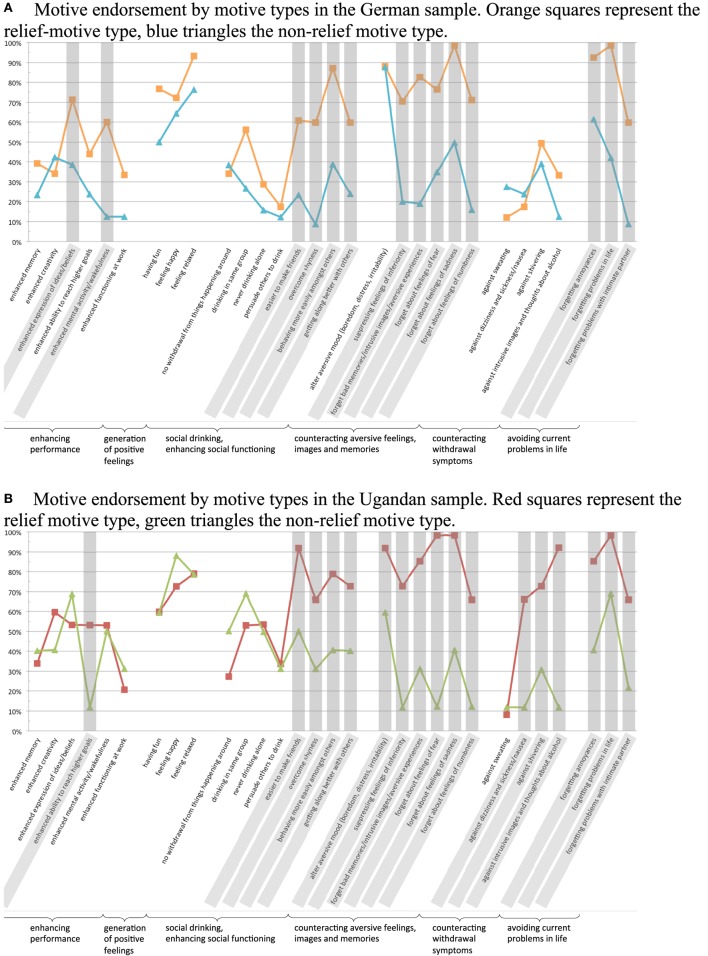
**(A)** and **(B)**: Motives, where conditional item probabilities differ by 30% or more between motive types are marked in gray.

The two separate LCAs yielded similar results. Two distinct motive types were found in both the German and Ugandan sample. One motive type was characterized by high endorsement of all items that are linked to negative reinforcement. Individuals in that group had a high relief motivation connected to alcohol. This became evident in the domain of social functioning, where overcoming insecurities in social interactions was especially relevant for the relief motive type. The same held true for overcoming aversive feelings, images, and memories and the suppression of current problems. Moreover, these same items were the ones that separated the distinct motive types in both samples (cf. gray markings in Figure 1). The only difference in the relief motive type between German and Ugandan dependent men was that the Ugandan men additionally strongly endorsed motives of drinking to relieve symptoms of withdrawal, whereas the German participants did not.

At the same time the relief motive type in both samples strongly endorsed drinking motive items connected to the positive reinforcement category “generation of positive feelings,” indicating that there is also a reward orientation in these individuals’ use of alcohol. However, the non-relief motive type in both samples endorsed items connected to the generation of positive feelings equally strongly. Further, the two motive types did not differ systematically in other reward-oriented items in both samples. Positive reinforcement through drinking in the sense of enhancing performance and having positive social interactions were not showing distinctively high or low conditional item probabilities in either motive type of any sample.

We classified dependent drinkers into motive types according to their maximum posterior probability. Average posterior probabilities were 0.99 for relief orientation in the German sample and 1.0 in the Ugandan sample. Posterior probabilities for non-relief orientation were 0.99 in the German sample and 1.0 in the Ugandan sample. The average posterior probabilities indicated very low classification errors. The LCAs grouped 18 German and 15 Ugandan dependent men in the relief motive group. Corresponding class probabilities were 41 and 60%. Consequently 26 German and 10 Ugandan dependent men were grouped into the non-relief motive group, with class probabilities of 59 and 40%.

### Comparison of Motive Types on Mental Health Relevant Characteristics

Across the 2 samples, 33 individuals were classified into the relief-motivated group and 36 into the non-relief-motivated group. The two motive types differed significantly in age, with the dependent men of the relief motive group being younger. Consequently, the onset of dependency happened at an earlier age for this group. Both differences were of medium effect size. The age at first encounter with alcohol was the same in both groups. Typical amount of consumption and alcohol-related symptoms were higher in the relief motive group, but differences only trended toward significance, with *p* = 0.06 and small effect sizes. Emotional, not physical childhood maltreatment, significantly differed between motive types. Members of the relief motive group reported more emotional maltreatment in their childhood. Finally, significant differences with medium to large effect sizes were found for psychopathological symptoms. The relief motive type suffered from more mental health-related problems as indicated by the general psychopathology composite score and the depression composite score.

Some indicators were assessed in one of the samples only. Traumatic events outside the family of origin including war events were assessed in the Ugandan sample only. Motive types did not differ concerning these events as indicated by small to negligible effect sizes. An exception was age at the time when the worst traumatic event had happened although this difference was non-significant (*r* = 0.25). Age was about 6 years younger in the relief-motivated group. Psychopathological symptoms (non-composite scores) showed relevant differences in both samples with the relief motive type being consequently more severely affected. Depression symptoms in the Ugandan sample (as measured by the DHSCL), general psychopathology in the German sample (as measured by the BSI), and problems with emotion regulation (as assessed by the DERS in the German sample) presented with medium to large effect sizes. However, the medium effect for symptoms of depression did not reach significance in the Ugandan sample due to the small sample size. Differences in PTSD symptoms in the Ugandan sample did not yield significant results.

## Discussion

Our results indicate that even though the treatment-seeking samples originate from diverse cultures and extremely different living contexts, the chronology of their addiction development seems to be similar. They reported first time consumption of alcohol consistently around age 14 years, the realization of dependency just before age 30 years, and similar chronicity of alcohol addiction. Moreover, they seem to have consumed approximately the same amounts of pure ethanol on a typical drinking day. In another treatment-seeking sample of German addicts, the same ages for first time consumption and the beginning of dependency have been reported (103). Kafuko and Bukuluki (104) conducted a qualitative research on knowledge, attitudes, and practices concerning alcohol with 30 focus groups in 5 different Ugandan districts and reported a matching age range of 12–15 years for first time consumption among boys.

### Motive Types and Their Alcohol Use

The LCAs resulted in strikingly similar patterns in drinking motivation. In each sample, a two-group solution provided both theoretical usefulness and the best data fit. The patterns of motive endorsement within these motive types were congruent across the samples up to the single motive item level. In brief, in both samples, reward orientation was similar across the motive types, whereas relief orientation was the motivation that separated the two motive types in both samples. Among both the German and Ugandan treatment-seeking addicts, one group stood out through their strong endorsement of motives representing negative reinforcement (relief motives); therefore, we called this group after its unique feature “relief motive type,” whereas motives representing positive reinforcement (reward motives) were about equally endorsed by both motive types in both samples.

The only difference between the German and the Ugandan sample were motive items related to avoidance of withdrawal symptoms. Both motive types endorsed these items in about equally low rates in the German sample, whereas in the Ugandan sample, all items except drinking to counteract excessive sweating were strongly endorsed by the relief motive type, but not the non-relief motive type. This difference may be a correlate of the significantly higher severity of alcohol-related symptoms in the Ugandan versus the German sample and more specifically in the Ugandan relief drinkers versus the Ugandan non-relief drinkers [mean AUDIT scores M = 30.4 (SD = 5.0) versus M = 25.3 (SD = 6.4), *p* < 0.05]. It may additionally be intensified by the fact that shortages in alcohol availability due to access or financial constraints are forcing Ugandan addicts to bear withdrawal symptoms more often than German addicts. Access to alcohol, and therefore the potential to consume at or even before the first hints of craving or withdrawal, is likely to be a given in the German sample at any time. Consequently, German addicts may not pursue motives connected to counteracting withdrawal. The fact that excessive sweating is the only relief-oriented item that is not differentiating between the Ugandan motive types may be due to the fact that sweating may not be recognized as a sign of withdrawal because it is omnipresent in every day life in Uganda and might be attributed to physical labor, climate, or frequent illnesses like malaria.

In agreement with the results of this study, other researchers have compared up to 13 countries and found cross-cultural consistencies in drinking motives as well. However, these studies have looked at exclusively non-treatment-seeking Western youths using factor analytic approaches. A four-factor model of drinking motivation has been confirmed in all countries, with the same ranking of youths’ drinking motive endorsement. Social motives were most frequent, followed by enhancement, coping, and conformity motivations (32, 49, 66, 68, 90).

In accordance with previous research findings, we did not find clear-cut coping, social and enhancement, or relief drinkers versus reward drinkers, as theoretical considerations according to the motivational model of alcohol use or the psychobiological model of craving for alcohol may imply (30, 34, 35). Relief drinkers in our sample also used alcohol to generate positive feelings and enhance their performance. This means that motives may change depending on specific situations or that several motives may be present within one drinking occasion. Although mostly validated in Western non-dependent adolescents and young adults, this result is in line with the literature that shows that drinking motives are not mutually exclusive, but highly correlated (32, 43, 45, 49, 58, 59, 66–68).

In agreement with these findings, Dvorak and colleagues (27) found that drinking motivations within individuals varied across drinking days in their longitudinal ecological momentary assessment study with young moderate drinkers. Crutzen et al. (55) examined an adult sample and also found significant correlations between motives. Finally Simpson et al. (59) investigated a clinical sample and reported that both enhancement and coping motives were highly endorsed simultaneously in individuals with comorbid alcohol use disorders and PTSD. The only study reporting on motives for drinking in a Ugandan population was a qualitative study analyzing data from 30 focus groups (half of them male and half female) in 5 districts, which included 2 northern Ugandan districts (Lira and Soroti) (104). The authors extracted drivers for drinking that matched the literature and our findings: social drinking, coping, escaping problems, overcoming boredom, stimulating the brain, and generating positive feelings. Under the heading “escaping from problems,” participants mentioned academic pressure, loss, the war, sicknesses and maltreatment, neglect, and communication problems in their families as antecedents of drinking. Drinking to overcome boredom was connected to unemployment and the lack of other recreational opportunities in the communities. Participants linked a stimulating effect of alcohol to enhanced mental activity and wakefulness and the ability to excel academically. Social drinking was reported to be connected to social functioning, e.g., being less shy and being able to face conflicts.

We found an approximate 2:3 ratio of motive type classification in both countries. In the Ugandan sample, we found 60% relief drinkers and 40% non-relief drinkers, whereas prevalence rates were almost the exact opposite in the German sample, with more participants being non-relief drinkers (59%). The German and Ugandan samples did not differ in the levels of general psychopathology and depression symptoms as measured by the established composite scores, i.e., differences in symptomatology do not explain the reverse prevalence rates. Furthermore, it is rather unlikely that the higher load of war-related and other traumatic experiences outside the family of origin explains the higher prevalence of relief drinkers in the Ugandan sample, since within Ugandan dependent men, relief motive drinkers did not report more traumatic experiences than non-relief motive drinkers, although events of forced perpetration were reported slightly more by relief drinkers. However, the difference was non-significant with a small effect size of *r* = 0.13.

Relief drinkers in our samples started drinking heavily significantly earlier and thus became addicts at around age 26, which was more than 6 years earlier than non-relief drinkers. This was also reflected by the fact that the relief drinkers in our samples were, on average, 8 years younger than the non-relief drinkers. They additionally drank slightly more (about four standard drinks) on a typical drinking day and consequently reported being affected more by alcohol-related symptoms. However, in this study, these differences did not reach significance and were mainly driven by the Ugandan sample.

Most of the literature examining drinking motives in dependent samples (59) or in youths find that enhancement and coping motives are both associated with measures of alcohol use, like quantity and frequency of intake, but that coping motives are especially linked to alcohol-related symptoms (32, 43, 66, 68, 88, 105). Lehavot et al. (58) reported a gender-specific effect for their sample of dependent drinkers. Coping motives predicted average alcohol intake for dependent women only, enhancement motives for both sexes. Our data fit well with these general results. The relief motive type following coping as well as enhancement motives was slightly, but not significantly, more affected than the non-relief motive type that basically followed enhancement motives only. Kuntsche et al. (68) found in their comparison of Canadian, Swiss, and North American youths that enhancement and coping motives predicted drinking frequency, quantity, and risky single-occasion drinking in all three countries, whereas conformity was reversely correlated and social motives did not predict alcohol use. Crutzen et al. (55) prospectively examined drinking motives in an adult population. Social motives at first measurement positively predicted number of drinks on the heaviest drinking day 3 months later, enhancement motives predicted the number of drinking days, and coping motives were positively associated with both measures of nonclinical drinking behavior. In sum, coping and enhancement motives have been most consistently linked to alcohol-related symptoms cross-sectionally (106) and longitudinally (107, 108), but mainly coping motives have been reported to be an important driver of the development of alcohol-related and addictive disorders in prospective investigations (57, 109).

Littlefield et al. (110) suggested that the longitudinal association between alcohol-related symptoms and enhancement motives might be a function of an overlap with coping motives. However, our data imply that clear-cut enhancement drinkers with little relief orientation also report high levels of alcohol-related symptoms. Especially in the German sample, AUDIT scores did not differ between the motive types [M = 23.7 (SD = 9.3) versus M = 22.4 (SD = 7.3)]. Wicki et al. (66) investigated adverse consequences beyond alcohol use or related symptoms in their sample of adolescents from 10 European countries using structural equation modeling. They found more adverse consequences for youths with higher intake linked to social, enhancement, and coping motives, on the one hand (indirect effects via intake), but also a direct link between coping motives and adverse consequences on the other hand. That is, coping motives lead to adverse consequences independent of alcohol use. In sum, the literature—but not necessarily our own research that focused on dependent drinkers—implies that certain drinking motives seem to weigh more than others in the development of alcohol-related disorders.

### Comparison of Motive Types on Mental Health Relevant Characteristics

Although our study neither draws on prospective information nor has the power to build more complex models incorporating multiple predictors to affirm interpretations, it seems that timely, more proximal traumatic events are not as relevant as childhood maltreatment in the prediction of who will become a relief drinker later in life. As opposed to other types of familial (physical maltreatment), war-related, or more general traumatic experiences, emotional maltreatment in childhood was especially closely connected to later drinking to alleviate aversive feelings and memories, symptoms of (social) anxiety, and current stress on top of an enhancement motivation. Similar results have been found by Potthast et al. (103) in their multiple regression models on alcohol-related outcomes: as opposed to physical maltreatment, sexual abuse, peer victimization, and other traumatic events, emotional maltreatment in childhood stood out as the most relevant predictor for age at onset of alcohol dependence and maximum lifetime drinking quantity.

Most of the few studies investigating traumatic experiences, drinking motives, and drinking behavior simultaneously were again conducted in young, mostly college student samples. Cross-sectionally, both drinking to cope and drinking to enhance mediated the association between students’ childhood trauma exposure and drinking behavior (105, 111, 112). However, Lindgren et al. (113) found in their prospective study with female college undergraduates that mediation was present for coping motives, but not for enhancement motives in the relationship between past sexual assault and alcohol-related outcomes. This means that the direct link between earlier traumatic experiences and drinking outcomes remains more relevant for enhancement drinkers than coping drinkers, which lead them to interpret their findings as supporting the self-medication hypotheses. Since the relief motive type is especially characterized by motive items connected to the use of alcohol to manage negative affect, it is not surprising that the relief drinkers in our samples significantly differed from the non-relief drinkers in emotional childhood maltreatment. With higher exposure to emotional maltreatment while growing up, it is likely that functional emotion regulation development is impaired, and individuals seek external means to control their emotions, such as the use of substances (114, 115).

Consequently, another correlate of being a relief drinker in our samples was suffering from more severe psychopathology. Differences between the relief motive type and the non-relief motive type were significant with medium to large effect sizes (cf. Table 2). In the German sample, the two motive types additionally differed significantly in emotion dysregulation. Deficits in emotion regulation or affect lability are often reported in the literature as being connected to both dysfunctional drug use and psychopathology (26, 116–121).

**Table 2:** Comparison of motive types on mental health relevant characteristics.

	Relief motive type (*n* = 33)	Non-relief motive type (*n* = 36)	Effect size (*r*)^a^	*p* value
Age	36.9 (9.7)	44.9 (10.8)	0.36	0.003
**Trauma exposure**				
*Childhood maltreatment*				
Physical maltreatment^b^	2.9 (2.1)	2.3 (2.2)	0.17	0.15
Emotional maltreatment^b^	4.3 (3.0)	2.9 (3.0)	0.25	0.037
*Traumatic events (VWAES)*				
Traumatic event types, experienced^c^^,^^d^^,^^e^	4.5 (2.1)	4.9 (2.3)	0.09	0.68
Traumatic event types, witnessed^c^^,^^d^^,^^e^	6.3 (2.3)	6.1 (3.5)	0.04	0.84
Traumatic event types, perpetrated^c^^,^^f^	0.5 (1.3)	0.2 (0.6)	0.13	0.51
Age at worst traumatic event^c^^,^^e^	26.0 (10.2)	32.1 (13.1)	0.25	0.21
**Alcohol-related indices**				
First consumption (age)	13.7 (5.0)	14.51 (4.2)	0.06	0.61
Start of dependency (age)	26.0 (7.4)	32.3 (11.2)	0.31	0.019
Chronicity of dependency (years)	11.7 (7.9)	14.0 (11.0)	0.07	0.59
Standard consumption on a typical drinking day^g^	22.3 (12.0)	17.9 (14.0)	0.22	0.06
**Psychopathology**				
Alcohol-related symptoms (AUDIT)^e^^,^^h^	26.8 (8.3)	23.2 (7.1)	0.23	0.06
Psychopathology composite	0.4 (0.9)	−0.3 (0.9)	0.43	<0.001
Depression symptoms score	0.5 (0.9)	−0.4 (0.9)	0.48	<0.001
PTBS symptoms (PDS)^c^^,^^i^	0.8 (1.5)	4.2 (7.1)	0.21	0.30
Depression symptoms (DHSCL)^c^^,^^e^^,^^j^	2.4 (0.6)	1.9 (0.8)	0.30	0.12
General psychopathology (BSI), GSI score^e^^,^^k^^,^^l^	1.6 (0.7)	0.8 (0.5)	0.53	<0.001
Emotion dysregulation (DERS)^e^^,^^k^^,^^m^	104.0 (28.3)	80.1 (20.5)	0.43	0.002

*If not otherwise indicated, results are based on nonparametric Mann–Whitney tests*.

*^a^Following Cohen’s suggestion effect sizes of r = 0.10 can be interpreted as small effect, r = 0.30 as medium effect, and r = 0.50 as large effect*.

*^b^Scale range: 0–10*.

*^c^Reported for the Ugandan sample only (*n* = 15 relief motive type, *n* = 10 non-relief motive type)*.

*^d^Scale range: 0–11*.

*^e^Results are based on a t-test*.

*^f^Scale range: 0–6*.

*^g^One standard drink contains 13 g of pure ethanol*.

*^h^Scale range: 0–40*.

*^i^Scale range: 0–51*.

*^j^Scale range: 1–4*.

*^k^Reported for the German sample only (*n* = 18 relief motive type, *n* = 26 non-relief motive type)*.

*^l^Scale range: 0–4*.

*^m^Scale range: 36–180*.

*AUDIT, Alcohol Use Disorders Identification Test; BSI, Brief Symptom Inventory; DERS, Difficulties in Emotion Regulation Scale; DHSCL, Depression-section of the Hopkins Symptom Checklist; GSI, Global Severity Index; PDS, Posttraumatic Stress Diagnostic Scale; VWAES, Violence, War and Abduction Exposure Scale*.

Notably, the German sample’s GSI score corresponded to a *T*-score of 72 according to the BSI manual’s norm tables for males. This means that the sample mean was well above the threshold for clinically significant symptomatology [cutoff ≥ 63 (97)]. Likewise, the Ugandan sample’s mean DHSCL score was ranging above the cutoff (≥1.75) most frequently used for judging clinical relevance of depression symptoms (96, 122–124). Although the levels of psychopathology remained in clinically relevant ranges for the non-relief motive type in both samples as well, they were significantly more pronounced in relief drinkers. The only measure that yielded results counter to our hypothesis was PTSD symptomatology assessed in the Ugandan sample only. However, we consider results on PTSD symptoms non-interpretable, since the general level of symptom endorsement was extremely low, with only eight men reporting symptoms at all. Our results are in line with findings reported by Simpson et al. (59) who found that links between psychopathological symptoms and alcohol consumption on the same day are more pronounced among coping drinkers than in individuals who ranked low on coping motives.

Cooper et al. (61) reported higher levels of coping motives in a sample of alcohol-dependent patients with comorbid social anxiety disorder than in alcohol-dependent patients without the comorbidity. For their adolescent samples in 10 European countries, Wicki et al. (66) concluded that individuals with more pronounced approach motives (enhancement and social) report higher subjective well-being as opposed to individuals with more pronounced avoidance motives (coping and conformity). They reported that coping drinkers seem to be especially prone to adverse consequences (e.g., injuries, academic problems, life dissatisfaction) beyond their alcohol use *per se*.

### Limitations and Conclusion

This study has several limitations that should be noted: the restriction to males and treatment-seeking individuals, the cross-sectional nature of our data, and our reliance on retrospective self-report. Due to the small number of women in our Ugandan sample, we decided to restrict our analyses to men only. Therefore, when interpreting the results, it has to be taken into account that important differences do not only exist in prevalence rates and drinking patterns between males and females but also exist in their experiencing of consequences, e.g., work and social functioning impairment has been reported to be most relevant in men, whereas intrapsychic problems resulting from drinking have been stressed in women (63). Lehavot et al. (58) also reported gender differences in the relationship between psychiatric comorbidity and drinking motives. In their study, PTSD severity predicted coping motives for both genders, but enhancement motives were predictive for males only.

In addition, our relatively small sample sizes have to be taken into account when interpreting the results of the LCAs. Although the classes were well separated, allowing for quite low sample size, and conceptual usefulness of the classes in practice was given, larger samples are warranted to validate our results. Moreover, larger sample sizes would allow for more complex multivariate analyses, e.g., logistic regression modeling to assess predictors of group membership simultaneously and structural equation modeling or other path analytic approaches to assess potential mediation and moderation in the interplay of trauma, emotional dysregulation, psychopathological symptoms, drinking motivation, and substance-related outcomes. Although this is far beyond the capabilities of our data, a distillation of the current literature would suggest a multiple mediated relationship between maltreatment or traumatic experiences and substance-related outcomes. Mediators that have been examined are emotion dysregulation and psychopathology that could also be modeled in a timely sequence. Drinking motivation could be considered as possible threefold moderator of the interplay: moderating the link between traumatic experiences and substance use [e.g., (113)], between emotion regulation and substance use [e.g., Ref. (25, 53)] and finally between psychopathology and substance use [e.g., Ref. (59)].

In sum, we can neither generalize our results to women and those experiencing lower degrees of or less chronic alcohol consumption or fewer alcohol-related symptoms nor those not seeking treatment. We had only few subjects abusing multiple substances in our sample and did not assess behavioral addictions. Both aspects might influence drinking motivation (125). Moreover, although we included antecedents of alcohol use (childhood maltreatment) and possible maintaining factors—in our case emotional dysregulation and psychopathological symptoms—that may activate drinking motives and lead to consumption, due to our cross-sectional assessment, we cannot interpret observed relationships causally. Reverse directionality may be possible or bidirectionality. Therefore, we reported differences between motive types only rather than speculating about associations of a specific directionality. Response bias is a general problem when relying on self-report. Therefore, we cannot rule out that our results could have been biased. However, it has been stated that self-report measures of substance use are largely valid (126). In future research, adding more objective measures of consumption, external assessment, and prospective designs, including ecological momentary assessment or intervention studies using random assignment, would be desirable to test hypothesized causal sequences.

Despite its limitations, this study is contributing to the sparse literature on drinking motivation in adult and alcohol-dependent samples and is one of the few concurrently assessing participant trauma history, drinking motivation, alcohol-related outcomes, and other psychopathologies. Moreover, it is the first study to address these issues in a non-western, conflict-affected sample. Using comparable methodology and measures in both countries allowed us to directly compare and aggregate data. We used LCA as a person-centered approach, since we were specifically interested in identifying potential motive subgroups of dependent drinkers and portraying their patterns of responding across single motive items.

Our results suggest two cross-culturally universal motive types within the larger group of chronic addicts. The two groups differed in relief motivation, not enhancement motivation. Individuals belonging to the relief motive type of drinkers were characterized by higher adversity in general, which supports construct validity. Although from diverse countries and contexts, the two samples of treatment-seeking chronic addicts showed striking homogeneity in addiction development, standard consumption, and contributing factors like childhood maltreatment and psychopathology. This implies that basic mechanisms and processes in the trauma, emotional dysregulation, psychopathology, drinking motives, and alcohol abuse relationship are comparable. However, we also showed that about 50% of alcohol-dependent men did not strongly endorse relief motives. This means a significant amount of alcohol addicts may not consider themselves as drinking to counteract current problems, withdrawal symptoms, aversive feelings, or memories and social insecurities. In future research, the LCA approach could be usefully implemented in more extensive cross-sectional designs, as well as longitudinal studies concerning alcohol use. For instance, to validate the results presented here, to investigate on characteristics that predict motive type membership, to find out whether membership predicts (long term) development of mental and physical health, and, finally, to assess whether the effects of prevention or intervention strategies vary across the motive types. The latter would ultimately inform motive type-tailored intervention strategies that may lead to improved prevention and treatment success, as well as higher intervention efficiency.

Wurdak et al. (127) evaluated a drinking motive-tailored intervention among adolescents admitted to hospital due to acute alcohol intoxication as a prevention strategy. They used a motive-tailored brief intervention combined with short online booster sessions and compared it to a general intervention based on motivational interviewing combined with alcohol-related information like the effects of alcohol intoxication and first aid strategies in short online booster sessions. In the motive-tailored group, enhancement drinkers, for instance, were suggested alternative ways of spending their free time and alternative ways to meet their need for sensation seeking. Coping drinkers were introduced to relaxation methods, they extended their problem-solving and life skills and learned ways to deal with stress. Bearing methodological limitations, such as a short follow-up period of only 4 weeks and a participant loss of 68% at follow-up in mind, the motive-tailored intervention proved to be superior to the active control intervention. However, effects were found for girls only, and boys profited approximately the same from both interventions. Apart form first efforts like these, intervention components tailored to systematically assessed motive types have not been evaluated scientifically in adult and dependent samples so far. The current literature suggests that at the same time factors such as gender, age, and comorbidities have to be taken into account (46, 58, 59, 127). In sum, research along these lines seems warranted and highly needed, given the scale of alcohol-related consequences for the affected individuals and their surroundings.

## Ethics Statement

Ethical approval for the studies was provided by the Ethical Committee of Bielefeld University following the guidelines of the German Psychological Society. These guidelines are in agreement with the American Psychological Association’s code of ethics. Additionally, all materials and procedures have been reviewed by the Lacor Hospital Institutional Research and Ethics Committee, Gulu, and approved by the Ugandan National Council for Science and Technology. All subjects gave written informed consent in accordance with the Declaration of Helsinki.

## Author Contributions

VE made substantial contributions to the conception and design of the work, the acquisition, analysis, and interpretation of the data; drafted, revised, and approved the manuscript; and ensures the accuracy and integrity of any part of the work. MP made substantial contributions to data acquisition, revised the manuscript critically for important intellectual content, approved the current version to be published, and agrees to be accountable for all aspects of the work to be accurate and integer. FN made substantial contributions to the design and interpretation of the data, revised the manuscript critically for important intellectual content, approved the current version to be published, and ensures the accuracy and integrity of any part of the work.

## Conflict of Interest Statement

The authors declare that the research was conducted in the absence of any commercial or financial relationships that could be construed as a potential conflict of interest.
